# Toward new gas-analytical multisensor chips based on titanium oxide nanotube array

**DOI:** 10.1038/s41598-017-10495-8

**Published:** 2017-08-29

**Authors:** Fedor Fedorov, Michail Vasilkov, Andrey Lashkov, Alexey Varezhnikov, Dirk Fuchs, Christian Kübel, Michael Bruns, Martin Sommer, Victor Sysoev

**Affiliations:** 1 Skolkovo Institute of Science and Technology, Skolkovo Innovation Center, 3 Nobel str., Moscow, Russian Federation; 2 V. A. Kotel’nikov Institute of RadioEngineering and Electronics of Russian Academy of Science, Saratov Branch, 38 Zelenaya str., Saratov, Russian Federation; 3 Yuri Gagarin State Technical University of Saratov, 77 Polytechnicheskaya str., Saratov, Russian Federation; 4 Institute for Solid-State Physics, Karlsruhe Institute of Technology (KIT), Hermann-von-Helmholtz-Platz 1, Eggenstein-Leopoldshafen, Germany; 5 Institute of Nanotechnology and Karlsruhe Nano Micro Facility, Karlsruhe Institute of Technology (KIT), Hermann-von-Helmholtz-Platz 1, Eggenstein-Leopoldshafen, Germany; 6 Institute for Applied Materials and Karlsruhe Nano Micro Facility, Karlsruhe Institute of Technology (KIT), Hermann-von-Helmholtz-Platz 1, Eggenstein-Leopoldshafen, Germany; 7 Institute for Microstructure Technology, Karlsruhe Institute of Technology (KIT), Hermann-von-Helmholtz-Platz 1, Eggenstein-Leopoldshafen, Germany; 8 Department of Functional Nanosystems and High-Temperature Materials, National University of Science and Technology “MISIS”, Leninskiy pr. 4, Moscow, Russia

## Abstract

Reliable environmental monitoring requires cost effective but highly sensitive and selective gas sensors. While the sensitivity of the sensors is improved by reducing the characteristic dimensions of the gas-sensing material, the selectivity is often approached by combining the sensors into multisensor arrays. The development of scalable methods to manufacture such arrays based on low-dimensional structures offers new perspectives for gas sensing applications. Here we examine an approach to produce multisensor array chips based on the TiO_x_ nanotube layers segmented by multiple Pt strip electrodes. We study the sensitivity and selectivity of the developed chip at operating temperatures up to 400 °C towards organic vapors in the ppm range. The results indicate that the titania nanotubes are a promising material platform for novel cost-effective and powerful gas-analytical multisensor units.

## Introduction

The development of gas sensors with advanced characteristics is driven by the necessity of fast, reliable and cheap monitoring of the environment^1^ which is especially important for various emerging tasks^2^. The most remarkable success in that field is expected in material science of gas-sensitive elements primarily matured from technologies which allow one designing nano-dimensional structures^3, 4^. The targeted sensor characteristics are higher sensitivity, stability and selectivity^5, 6^ which should be combined with low production cost and low energy consumption of the final units^6–8^.

So far, metal oxide semiconductors are recognized as materials which fully meet the requirements of commercial gas sensors and have a great potential to advance further, mainly through application of nanotechnologies^9^. The general approach to greatly enhance their sensitivity is a reduction of the characteristic dimensions of the crystallites/grains composing the material^10^. This enhances the impact of surface processes on the total conductivity of the material as the ratio between Debye length and structure size increases^11^. However, 3D-nanosized oxide structures might be rather unstable especially at elevated temperatures which are conventionally employed to activate the sensors, particularly chemiresistors^12^, what questions their stability. In order to address this issue, quasi-1D structures such as nanowires, nanobelts or nanotubes (NTs) are considered to be the promising candidates for a new generation of gas sensors (see recent reviews^13–15^ and references therein). Still, the selectivity remains to be the most challenging aspect because metal oxide semiconductors are fundamentally unselective for chemisorbed gas species. Therefore, the most powerful way is to combine various sensors^16^, including those based on quasi 1D structures^17^, into arrays and process the obtained vector signal by pattern recognition techniques.

The quasi 1D oxide structures can be synthesized via gas-based or solution-based techniques^18–21^. The second one is thought to be more suitable for mass production because of lower cost and better control the surface area to define the thickness at the nanoscale^22^. Among quasi-1-D structures, NTs are distinguished by their high specific surface due to their hollow structure with inner and outer surfaces that are exposed to the gases which makes it easier to fully deplete the oxide from free carriers due to surface localization under chemisorption and catalytic processes. That favors a “transduction function” in this material for sensor applications while a “receptor function” is still defined by a preferential oxide crystalline phase^20, 23^.

In this report, we consider NTs of titanium dioxide which can be reliably fabricated using high yield protocols^24^. The most cost effective and well established technology is anodization of titanium in F^−^containing electrolytes which is appropriate for mass-scaling^20^. By properly choosing the anodization bias one can tune fabricating oxide NT layers with precise control of the diameter, height, wall thickness of the tubes, inter-tube distance, thickness of the barrier layer, etc.^25^. This renders the material to be promising for chemiresistors using a conventional two-electrode design^26–30^. Although, a realization of these sensors is challenged by a negative influence of titanium underlayer which shunts NTs and leads to poor electrical contacts which reduces the reliability of the devices^31^.

Despite some success in enhancing the sensitivity of the TiO_2_ NT-based sensors, the selectivity of gas identification remains unresolved. Even the approaches to functionalize the NTs by various dopants have not allowed one obtaining absolute sensitivity to only one gas^32, 33^. Therefore, here we consider employing a multisensor approach^34^ to be realized possibly in the cheapest design^35^ able in frames of microelectronic technologies by segmenting the pristine NT array by multiple co-planar electrodes. In particular, we demonstrate the capability of the chip to selectively detect few exemplary organic vapors at ppm concentrations.

## Results

**Figure 1:**
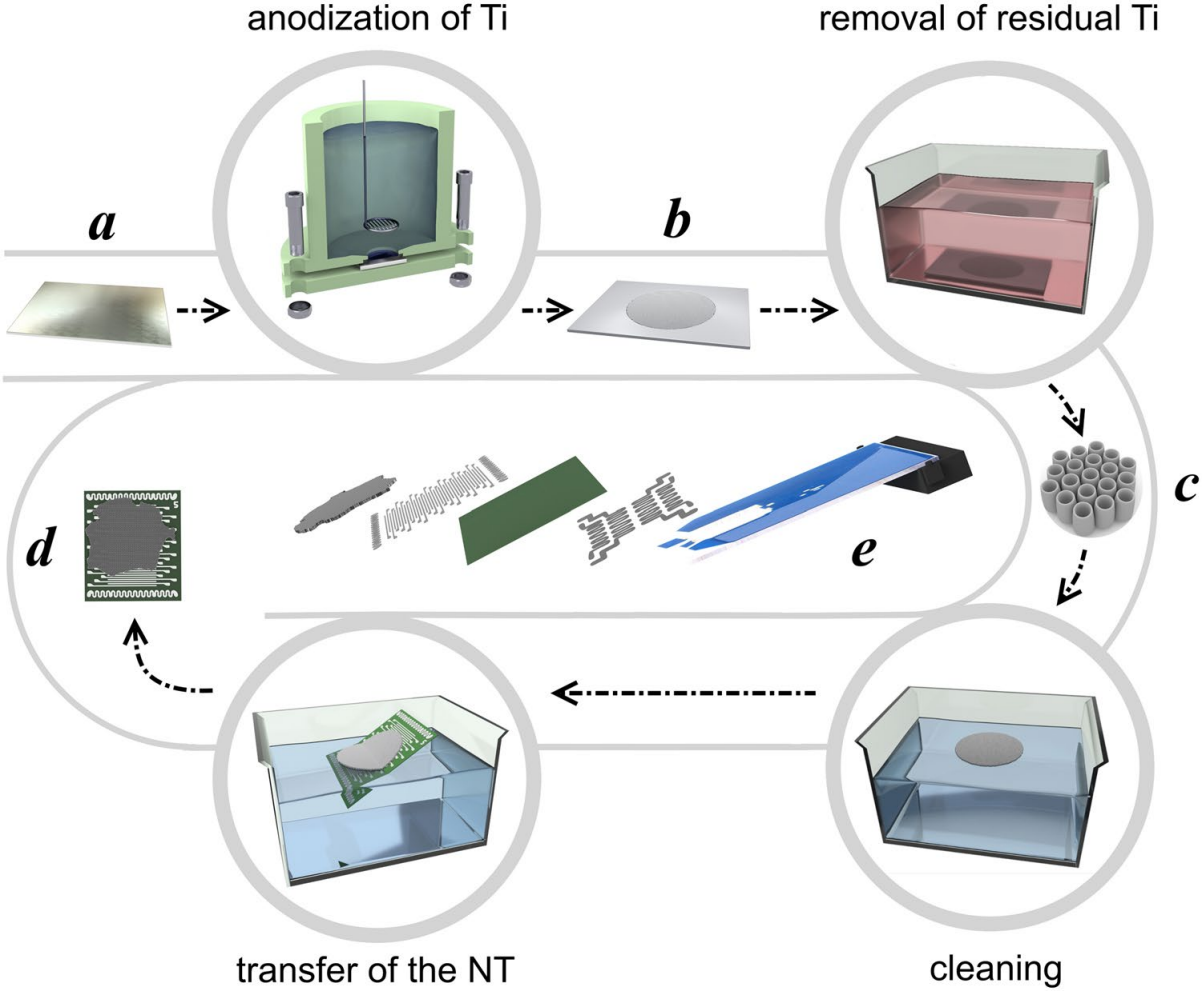
The scheme of fabrication the multisensor chip based on TiO_x_ NT array: (**a**–**e**) denote respectively Ti foil (**a**), Ti foil with as-prepared NT array (**b**), the extracted NT array (**c**), the NT array placed over the chip (**d**), the scheme of multisensor chip wired into 50-pin ceramic card (**e**).

The process to employ the titanium dioxide NTs in the multisensor chip includes (Fig. 1) primarily an anodization of the titanium foil in accordance with known protocols^20, 25, 36, 37^. The as-anodized samples are further etched for removing the remained metal in absolute methanol/ Br_2_ solution^38^ in order to extract the pristine TiO_x_ NT array. The full details of the processes are given in Methods. Then, the TiO_x_ NT array is cleaned in pure alcohols and placed for floating over the distillated water surface. This array is then picked up and thoroughly positioned over the Si/SiO_2_ multielectrode chip substrate^39^. The as-prepared chip is dried for few hours at room temperature in air.

The Si/SiO_2_ chip substrate equipped with multiple Pt strip electrodes provides a number (up to 38) of NT array segments whose electrical/chemiresistive properties are measured independently.

**Figure 2:**
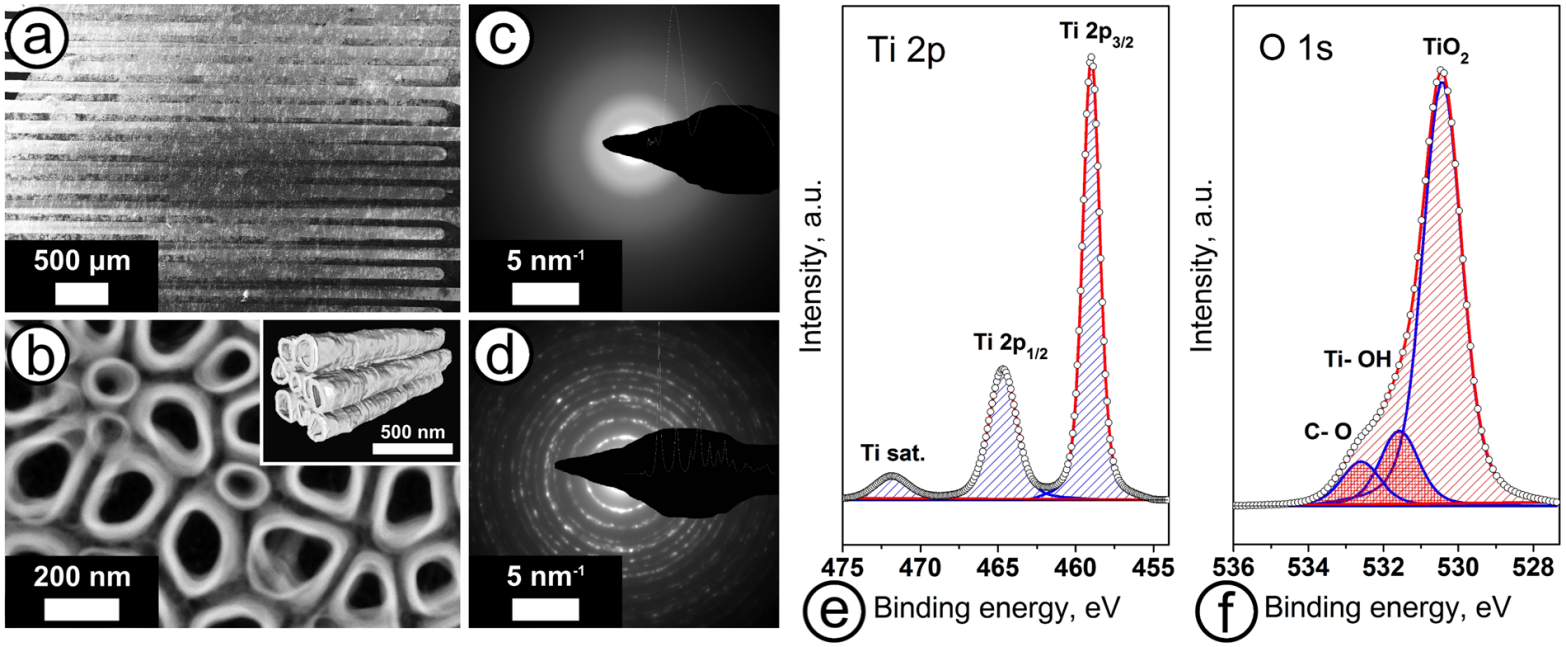
Characterization of the TiO_x_ NT arrays used in the multisensor chip: (**a**) SEM image taken of the chip surface covered with NT array; (**b**) HAADF-STEM image, the inset shows a surface rendering of the 3D STEM tomographic reconstruction viewed along the NT length; (**c**,**d**) SAED patterns of as-grown NTs and NTs annealed at 400 °C, respectively; (**e**,**f**) the XPS peaks related to Ti 2p (**e**) and O 1s (**f**) for the as-grown NTs, red lines show the envelope curves of all fitted peaks to compare with the experimental data (open circles).

Following the transfer into the chip the TiO_x_ NT array possesses a structural integrity as evidenced by scanning electron microscopy (SEM) inspection (Fig. 2a); the NTs are characterized with a distinct morphology (Fig. 2b).

The NTs are stand vertically to be almost perpendicular to the chip surface that means the NT open endings are positioned towards the outer atmosphere, including gas vapors under exposure. The mean NT inner diameter *D* and the mean wall thickness *d* are 102.7 ± 18.1 nm and 14.5 ± 5.2 nm, respectively, while the NT density is ca. 4.3·10^9^ cm^−2^. The SEM imaging of the array layer cross-section has proved the height of the NTs to be in range of 0.9–1.2 μm. This height ensures an easy gas access to NTs surface and according to Albu *et al*.^40^ allows one staying adhered to chip surface and free of cracks under heating to temperatures higher than 300 °C which are applied for the activation of chemiresistive effect in the NTs and chip operation.

In order to clarify the structure and morphology of NTs we have extracted the NT array out of the same batch as the ones employed in the chip for additional characterization by high-angle annular dark field-scanning transmission electron microscopy (HAADF-STEM) and X-ray photoelectron spectroscopy (XPS) techniques; the details are given in Methods.

Moreover, we have analyzed NTs in as-grown state and one following annealing at 400 °C for 24 h in air atmosphere in order to simulate conditions of chip operation. The recorded HAADF-STEM images of both kinds of samples, as-grown and annealed ones, show that the TiO_x_ NTs are closely packed into an array where adjacent NTs have a distance of a few nm, which favors good gas access (Fig. 2b and Supplementary). The annealing at 400 °C does not change the tubular structure of the arrays. The 3D STEM tomographic reconstruction of the whole array indicates the existence of interconnections between NTs along their whole height (see inset at Fig. 2b and Supplementary videos). Some of the NTs bifurcate that increases their connection pathways and specific surface area. Selected area electron diffraction (SAED) supports that the TiO_x_ NTs are mostly amorphous in the as-grown state (Fig. 2c) to be organized into polycrystalline structure at anatase TiO_2_ phase after the annealing (Fig. 2d). The anatase crystal structure of annealed NTs is manifested by lattice strong reflections of (101), (103), (105) planes corresponding to d-values of 3.52 Å, 2.37 Å, 1.75 Å and reflections from other crystal planes (see Supplementary).

The XPS study of the as-grown NT array placed over Au-coated Si/SiO_2_ substrate yields that Ti 2p_3/2_ peak is positioned at 459.0 eV and the corresponding O 1s peak is observed at 530.3 eV, both in a good agreement with literature references^41^. Surface Ti-OH groups are indicated by the O 1s peak at 531.2 eV which cannot be seen in the energy region related to Ti 2p due to a low intensity. The weak higher binding energy O 1s peak at 532.4 eV corresponds to C-O groups and corroborates with C 1s signal from surface contaminations component observed at 289.1 eV (Fig. 2e, Supplementary). The XPS survey spectrum also suggests the presence of fluorine traces in the as-grown NTs which stem from electrolyte residues and are removed after annealing of NTs at 400 °C in accordance with literature^42^. Still, other studies support possible fluctuating of the foreign elements, [F] and [C], at low concentrations in NTs^43^ and the existence of Ti in different oxidation state close to Ti/TiO_2_ interface^44^. Such minor variations of structure and elemental composition in NTs have been out of XPS detection in our samples but still could contribute to the observed differences in local electrical and gas-sensitive properties along NT array membrane as further discussed.

Based on the relative intensity of the Ti 2p_3/2_ and O 1s peaks, the surface [O]/[Ti] atomic ratio is calculated to be about 1.92. The annealing of NTs at 400 °C in air increases the oxygen content towards attainment of the stoichiometry ratio equal to approx. 1.95. Obviously, even in annealed state the TiO_x_ NTs appear to be non-stoichiometric with an oxygen deficiency which is a major prerequisite to observe a chemiresistive effect^45, 46^.

**Figure 3:**
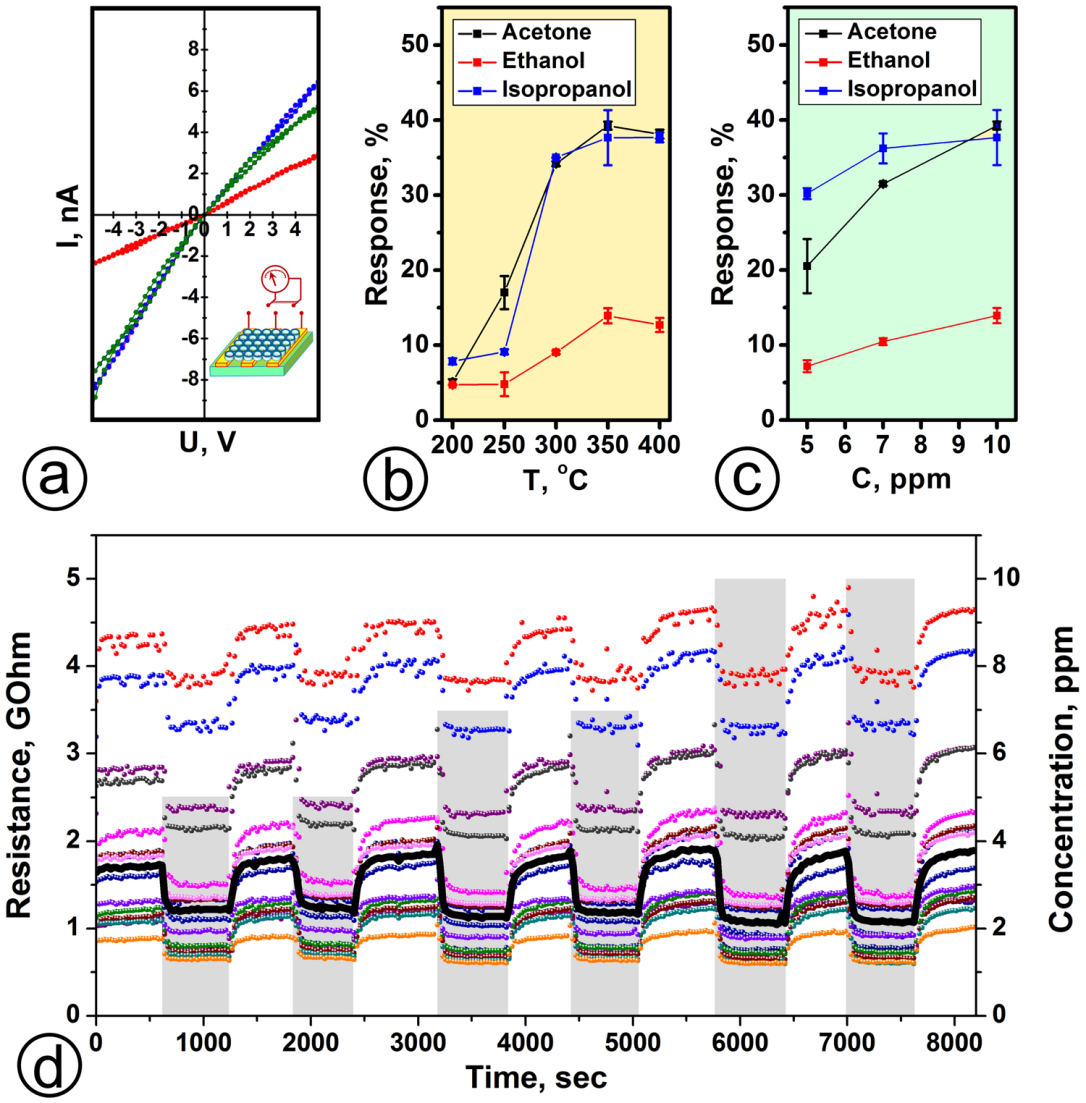
The electrical characterization of TiO_x_ NT array based multisensor chip: (**a**) I–V curves recorded from three exemplary segments under constant flow (100 sccm) of laboratory air at 350 °C; (**b**) the dependence of chip segments response to 10 ppm of ethanol, isopropanol, and acetone on operating temperature; (**c**) the dependence of chip segments response on vapor (ethanol, isopropanol, and acetone) concentration in air at 350 °C; (**d**) the variations of the chip segments resistances, *R*(*t*), to pulses of isopropanol at different ppm concentrations under operating temperature of 350 °C; thick black line denotes the segments median resistance.

In order to activate and stabilize the as-prepared NTs^47^ for gas sensing tests, we have primarily heated the NT-based multisensor chip up to 400 °C for 24 h in air atmosphere and then carried out the isothermal electrical measurements in RT-400 °C range. At operating temperatures lower than 200 °C the NT array segment resistance over the chip was too high to be read out by our electronic measuring units (see Methods). In the temperature range of 200–400 °C, the I-V curves measured for the NT array segments have been found to be nearly linear (Fig. 3a) in air and in the test vapors (isopropanol, ethanol and acetone) mixed with air. This reasonably indicates a good contact between NTs and the electrodes without significant Schottky barriers. A temperature increase from 200 °C to 400 °C leads to a drop of the mean resistance of the NT array segments in air from tens of GOhms to hundreds MOhms (see Supplementary) following a semiconducting behavior. The activation energy calculated from the conductance-vs-temperature curve is around 0.44 eV which correlates well with the reported values for polycrystalline TiO_2_^48^. This value originates from the potential barriers between adjacent NTs^49^ and/or the energy levels of local states related to oxygen vacancies^45, 46^ in the oxide gap which are involved in electron exchange processes at the NT surface.

Following the chip exposure towards isopropanol, ethanol and acetone vapors, 5–10 ppm concentrations, in mixture with laboratory air at 30–35 rel. % humidity, we have observed a clear chemiresistive effect in NTs at all operating temperatures in 200–400 °С range. The maximum gas response as a relative resistance change, *ΔR/R*_*air*_, in the vapor-enriched air has been observed at 350 °C (Fig. 3b). At this temperature, the median NT array segments response to isopropanol and acetone of 10 ppm concentration is close to 38% while one to 10 ppm of ethanol is about 14%. The concentration dependence of the gas response at this temperature follows Freundlich isotherm, *ΔR/R*_*air*_ *= kC* ^*n*^, where power low index *n* for isopropanol, ethanol and acetone is about 0.4, 0.9, 0.6, correspondingly (Fig. 3c) which is quite typical for the most metal oxides^50^. The observed signals have been stable, at least for few days (see Supplementary).

These remarkable differences in responses to these gases are caused by variations in chemical adsorption and dissociation of these molecules at the NT surface that provides a background to selective recognition of these vapors by multisensor chip array as further discussed. Here, it is worth noting that the change of the median resistance exceeds 8% to 5 ppm of isopropanol already at 200 °C what indicates rather low gas detection limit for the developed multisensor. Figure 3d shows the typical reduction of resistance of exemplary segments of TiO_x_ NT array when exposed to isopropanol vapors at 350 °C which is optimal one to observe the chemiresistive effect. The similar data have been observed in case of other test vapors at different temperatures.

**Figure 4:**
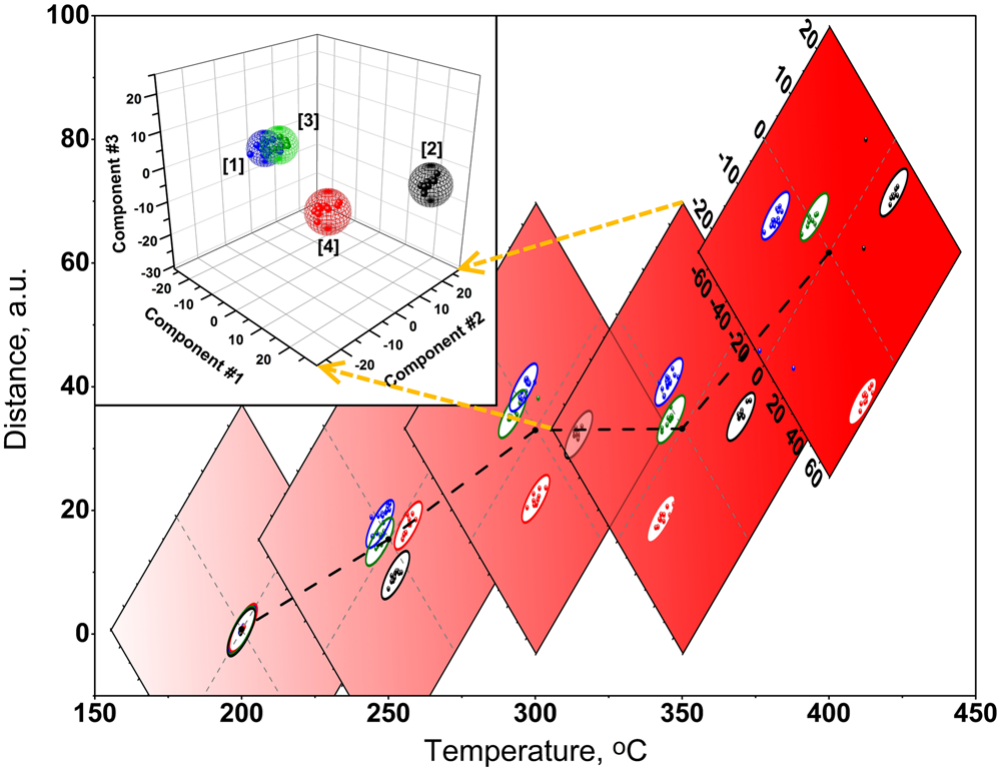
The LDA processing of vector responses of NT array-based multisensor chip to test organic vapors, 10 ppm concentration: dependence of the average Mahalanobis distance between the centers of vapor-related LDA clusters as a function of operating temperature. The projections of the generated LDA space into primary two-component plane are centered at the obtained points referring to Mahalanobis distance. The inset is the LDA 3-component diagram built for the chip operated at 350 °C: 1-air,2-acetone,3-ethanol,4- isopropanol. All the spheres are calculated at 0.95 confidence level to frame data training clusters; the points are related to the test responses.

It is clear that the organic vapor traces substantially decrease the resistance of the segments in accordance with *n*-type semiconductor response of non-stoichiometric TiO_2_. Still, the spatial inhomogeneous local morphology of the NT array across the chip segments results in the variation of both the resistance and gas sensitivity. It allows one employing it to build a vector signal whose components are constitued by signals from the gas-sensitive segments in the chip and approach the gas selectivity in the framework of known multisensor concept^16^. In order to process the NT-based multisensor vector response to the test vapors we have applied the Linear Discriminant Analysis (LDA) which maximizes the vector variations induced by different vapors and minimizes the vector scatters under exposure to certain vapor^51^. The confidence probability to build the LDA clusters related to gases has been installed to be equal to 0.95. By these means, we have tested all the multisensor chip vector signals recorded at operating temperatures in 200–400 °C range (Fig. 4). In order to build the training set in the LDA space the sampling of 10 vector signals recorded under each atmosphere has been employed while other recorded signals were served for testing. Here, we define the selectivity as an average Mahalanobis distance between clusters in the LDA space^52^. This parameter allows us to estimate quantitatively the quality of gas recognition under various working conditions of the chip. As we found, the selectivity strongly depends on the operating temperature (Fig. 4b,c). The temperature enhancement from 200 °C to 400 °C results in 2-order change in the mean Mahalanobis distance between the clusters from ca. 0.8 to 61.7, correspondingly, in the 3-dimensional LDA coordinate system. This selectivity estimation clearly correlates with gas response behavior with temperature up to 350 °C: the higher response means higher selectivity. However, 400 °C is even more favorable for selectivity while gas response slightly reduces. These data indicate that the operating temperature facilitates not only the at-surface charge-exchange processes but also the minor variations of potential barriers observed in NT-to-NT percolation paths in the array.

While the electron exchange between the chemisorbed species and semiconducting oxide surface depends mostly on the material itself, the micro- and mesostructure of the layer, its porosity and characteristic geometry substantially contribute to the overall NT array resistance measured at different environments^53^. Basically, the two dominant fundamental mechanisms, the depletion of the oxide material by free electrons and existence of potential barriers in contacts between the NTs, interplay in the chemiresistive response of the NT array. Under air conditions the NT surface is fully covered by oxygen and hydroxyl chemisorbed species which localize the electrons from the oxide conduction band^54^. This induces at-surface space charge region whose width in the oxide is comparable with the average NT wall thickness and crystallites. Taking the obtained NT array resistance values measured in air conditions we assess^55^ the free electron concentration, $n=\frac{g}{e\mu h{R}_{s}}$, where *e* is elementary charge, *h* the tube height, *R*_*s*_ the NT array sheet surface resistance per square (see Supplementary), *μ* the electron mobility, which we consider equal to approx. 0.1 cm^2^/Vs, characteristic for polycrystalline TiO_2_^56^. The geometry coefficient $g=\frac{{(D+2d)}^{2}}{2d(D+d)}$ accounts for reduction of in-fact volume of the NT array sheet when compared to compact layer due to the porous tubular geometry and confining the electron pathway within the NT walls. Considering the obtained magnitudes of *D* and *d* the estimated value of *g* is equal to approx. 5.1. For instance, at operating temperature of 350 °C one gets *n* equal to approx. 3.3 ± 0.4·10^13^ cm^−3^ that is lower by 3–4 orders of magnitude when compared to concentrations reported for a single anatase NT measured in vacuum conditions^57^. The corresponding Debye length, ${L}_{d}=\sqrt{\frac{\varepsilon {\varepsilon }_{0}kT}{{e}^{2}n}}$, where *k* is Boltzmann constant, *ε*_0_ the electric constant, *ε* the dielectric permittivity of anatase (equal to approx. 31)^58, 59^, *T* the operating temperature, at these conditions goes to micrometer range that obviously exceeds the NT wall. Therefore, under air conditions the Fermi level in the NTs is pinned at the local states induced by oxidizing/hydroxyl adspecies in the oxide gap that is ordinarily described as a flat-band approximation^60^. It results in remarkable reduction both the free electron concentration in the NT wall and the electron mobility due to enhancing the potential barrier, *ψ*_*B*_, between NTs^61^. Consequently, this originates a high NT array resistance observed. So, the electron transport in such structures is mainly governed by carrier-depleted NT walls contacting each other like in the case of grains in polycrystalline samples^54^.

**Figure 5:**
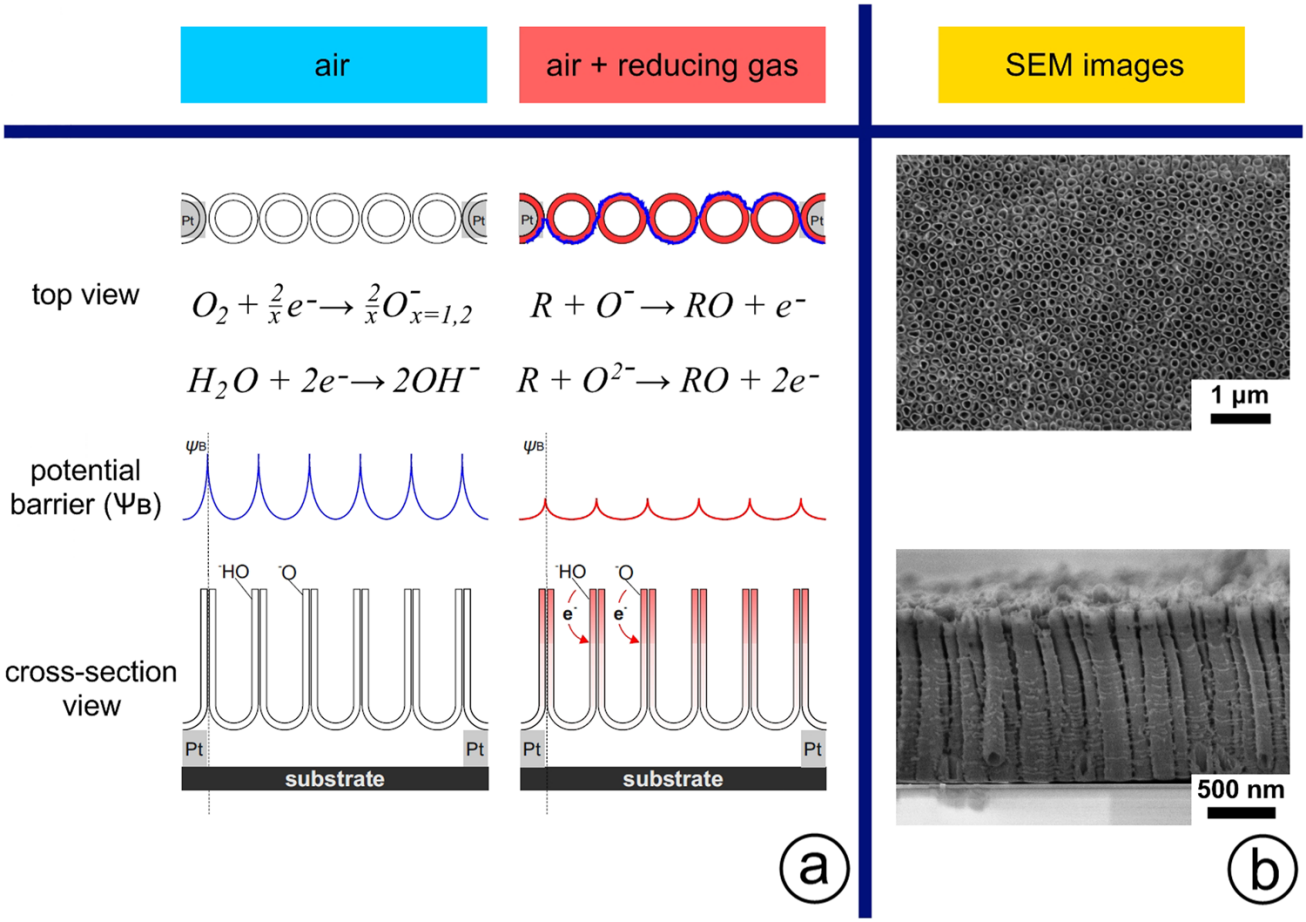
A schematic representation to show the change in the conductivity and conduction pathway for electrons through TiO_x_ NT array at two environments - air and mixture of air and reducing agents: (**a**) the modification of potential barrier heights following the adsorption of the species of different origin and their surface reactions; (**b**) SEM images of the NT array from top and side views corresponding to the presented scheme.

The exposure to reducing organic vapors releases the at-surface localized electrons to conductance band and decreases *ψ*_*B*_^50, 55^. We consider the only electronic component of the oxide conductance because the operating temperatures below 400 °C are still not enough to stimulate a significant exchange between surface and bulk of NTs by intrinsic defects^58^. So, under application of electric field the electrons go through NT adjacent walls whose potential barrier height and “tunneling density” depend on a gas environment (Fig. 5). Thus, the whole NT array looks like a network of percolating tubes where analyte molecules modify the conduction pathways upon interaction with NT surfaces and junctions. The surface reactions between the gas molecules (see Fig. 5a) and/or their direct catalysis include a dissociation accompanied by an exchange of charged species that involves different amounts of charge localized by chemisorbed species.

This explains the higher response of the NTs towards isopropanol and acetone vapors when compared to one *versus* ethanol. Moreover, a stochastic combination of NT-to-NT contacts at each chip segment underlies the diversity of their gas-sensing properties along the array that allows us to build the chip vector signal selective to gases.

*In conclusion*, the obtained data clearly show that TiO_x_ NT array is a promising material to employ not only for the development of conventional gas sensors but gas-analytical multisensor chips as well. We have introduced the approaches to build up such chips which include primarily an anodization of titanium into oxide nanotubular layer with following removal of residual metal and secondly the transfer of the layer over the multielectrode Si/SiO_2_ chip substrate by a method similar to Langmuir-Blodgett one. The tested samples of NT array- based multisensor chip have exhibited high sensitivity towards organic vapors in ppm range. The intrinsic stochastic inhomogeneities of the array along the chip yield obtaining the selectivity of gas discrimination by processing the multisensor vector signal generated by chip segments via pattern recognition technique. Still, the chip design allows one further advancing the selectivity by application of inhomogeneous heating and/or other external modifications.

## Methods

### TiO_x_ NT array fabrication

TiO_x_ NTs are prepared by anodization of Ti, 99.6% (Sigma-Aldrich) foil of 127 µm thickness. Prior to anodization the titanium foil has been immersed into acetone and ethanol, both for 10 min, in sequence under sonification and then rinsed in deionized water (18.2 MΩcm). The titanium foil is characterized with a roughness less than 0.7 µm measured with surface profiler (Veeco DekTak 150). The electrochemical anodization has been performed in a Teflon® cell in two-electrode configuration with a platinum mesh as cathode biased at 30 V for 1 hour at room temperature using electrolyte based on glycerol/water 3:1 solution with addition of 0.8% NH_4_F (Supplementary Figure 1). All chemicals have been provided to be at least of analytical purity (Sigma-Aldrich). The electrode area is separated by O-ring rubber to expose a Ti surface, 0.79 cm^2^, to the electrolyte. The anodization experiments have been carried out using a high-voltage potentiostat (Keithley 2410 source meter) connected to a digital multimeter (Keithley 2700 multimeter /data acquisition system) interfaced to personal computer (PC). The TiO_x_ NT array is afterwards rinsed in ethanol and is dried at room temperature for 0.5–1 h. The Ti sublayer remains have been selectively dissolved in an absolute methanol/Br_2_ solution, 1:9 vol., at 20 °С for 2 hours according to ref. 38. Then, the obtained layer has been positioned over the Si/SiO_2_ chip equipped with multiple planar electrodes. Thermal treatment of titania NTs has been performed in PEO-601 oven (ATV Technologie GmbH) in air atmosphere at 400 °C during 24 h. Following the annealing the samples have been rinsed by distilled water and dried in air.

### TiO_x_ NT array characterization

The morphological characterization of the NT array has been carried out using a scanning electron microscope (AURIGA® CrossBeam® workstation, Carl Zeiss) equipped with an energy dispersive X-ray (EDX) detector. The NT structure has been studied by transmission electron microscopy (TEM) using an aberration (image) corrected Titan 80–300 (FEI Company) equipped with a US1000 (Gatan) CCD camera. After mechanical removing the TiO_x_ NTs from the substrate, they have been dispersed in *n*-heptane by sonification and deposited on a carbon coated Cu grid (Quantifoil) for the TEM analysis. For an electron tomographic characterization in HAADF-STEM mode, 15 nm gold nanoparticles (University of Utrecht) were added for alignment and a tilt-series recorded from −60° to 60° in 2° steps. The tilt-series alignment was performed using IMOD (University of Colorado) and the 3D volume reconstructed using the SIRT algorithm implemented in Inspect3D (FEI Company). The Supplementary video shows a volume rendering of the 3D structure of the NT array morphology and a surface rendering of a selected part of the 3D reconstruction prepared in Amira (FEI Company).

The X-ray photoelectron spectroscopy (XPS) measurements have been conducted using a K-Alpha+ XPS spectrometer (Thermo Fisher Scientific, East Grinstead, UK). Data acquisition and processing using the @Thermo Avantage software are described elsewhere^62^. We have utilized Si substrate with sputtered Au layer, approx. 1 µm thick, to place the titania NT array after the metallic Ti is removed. All samples have been measured using a microfocused monochromated Al Kα X-ray source of 30–400 µm spot size. The K-Alpha charge compensation system is employed during analysis using electrons of 8 eV energy and low-energy argon ions to prevent any localized charge build-up. The spectra are fitted with one or more Voigt profiles; the binding energy uncertainty is ±0.2 eV. The analyzer transmission function, Scofield sensitivity factors^63^ and effective attenuation lengths (EALs) for photoelectrons are applied for quantification. EALs are calculated using the standard TPP-2M formalism^64^. All spectra have been referenced to the C 1s peak of hydrocarbon at 285.0 eV binding energy controlled by means of the well-known photoelectron peaks of metallic Cu, Ag, and Au.

### The multielectrode substrate as a platform for the multisensor chip

We have utilized multielectrode chip based on Si/SiO_2_ substrate equipped with 39 Pt strip electrodes which define, if necessary, 38 segments of the NT array as a maximum number^39^. The inter-electrode distance is about 80 µm, electrode width is 100 µm, and the electrodes are 1 μm thick. All the electrodes have a length of about 4 mm that defines the surface area of about 4 × 8 mm^2^ whose part or total is covered by NT array. The frontside of the substrate has two meander-shaped Pt thermoresistiors at the edges to monitor the operating temperature which is adjusted by four meander heaters located at the backside. The operating temperature is maintained by corresponding PC-driven electronics unit with accuracy of 1 °С while the temperature distribution at isothermal chip operation can be of ±10 °C^52^.

We have tested a number of chips in preliminary studies, the reported data belong to one exemplary chip.

### Gas response measurements with NT array multisensor chip

The response of multisensor chip equipped with TiO_x_ NT array towards different organic vapors has been studied using a setup which includes home-made data acquisition unit, test gas generator, gas delivery tubes and electronic unit to maintain and monitor the chip operating temperature. Data acquisition module (National Instruments USB-6259) together with current pre-amplifier (SRS, SR570) has provided the resistivity measurements up to tens GOhms of each segment in the chip. To read out the total array resistances we have employed a multiplexing card for switching between the segments. Test vapor mixtures with laboratory air have been obtained with gas generator (Owlstone, UK) which employs heated permeation tubes containing a test liquid to yield vapors of several ppm of concentration under equilibrium conditions. The vapors of the same reducing origin, alcohols and acetone, have been utilized as test ones. The vapors have fed the chip by series of 10 min duration interrupted by pulses of pure laboratory air for 10–25 minutes to ensure the recovery of the chip segment resistances. The concentration of vapors in mixture with air has been maintained to be 5, 7, 10 ppm for each one. The laboratory air is delivered by an oil-free compressor as is to be of normal humidity level in range of approx. 30–35 rel. %. The all gases have been supplied in a flow mode with 100 sccm rate. We have utilized only the data for NT array segments with resistivity less than 10 GOhm at maximum operating temperature of 400 °C. Therefore, 16 segments of the multielectrode chip have been considered in the reported data.

## Electronic supplementary material

Supplementary Info

Video 1

Video 2
