# Supplementary material for: Toward new gas-analytical multisensor chips based on titanium oxide nanotube array
Source: Sci Rep. 2017 Aug 29;7:9732. doi: 10.1038/s41598-017-10495-8 (PMC5575241; doi:10.1038/s41598-017-10495-8)
Supplement: Supplementary file 1 — Supplementary Info [file 41598_2017_10495_MOESM1_ESM.pdf]

Supplementary Information to the Paper

"Toward new gas-analytical multisensor chips based on titanium oxide nanotube array"

by

Fedor Fedorov[1], Michail Vasilkov[2,3], Andrey Lashkov[3], Alexey Varezhnikov[3], Dirk Fuchs[4], Christian Kübel[5], Michael Bruns[6], Martin Sommer[7], Victor Sysoev[3,8*]*

[1] Skolkovo Institute of Science and Technology, Skolkovo Innovation Center, 3 Nobel str., Moscow, Russian Federation

[2] V. A. Kotel'nikov Institute of RadioEngineering and Electronics of Russian Academy of Science, Saratov Branch, 38 Zelenaya str., Saratov, Russian Federation

[3] Yuri Gagarin State Technical University of Saratov, 77 Polytechnicheskaya str., Saratov, Russian Federation

[4] Institute for Solid-State Physics, Karlsruhe Institute of Technology (KIT), Hermann-von-Helmholtz-Platz 1, Eggenstein-Leopoldshafen, Germany

[5] Institute of Nanotechnology and Karlsruhe Nano Micro Facility, Karlsruhe Institute of Technology (KIT), Hermann-von-Helmholtz-Platz 1, Eggenstein-Leopoldshafen, Germany

[6] Institute for Applied Materials and Karlsruhe Nano Micro Facility, Karlsruhe Institute of Technology (KIT), Hermann-von-Helmholtz-Platz 1, Eggenstein-Leopoldshafen, Germany

[7] Institute for Microstructure Technology, Karlsruhe Institute of Technology (KIT), Hermann-von-Helmholtz-Platz 1, Eggenstein-Leopoldshafen, Germany

[8] Department of Functional Nanosystems and High-Temperature Materials, National University of Science and Technology "MISIS", Leninskiy pr. 4, Moscow, Russia

-

E-mail address: fedorov_fs@daad-alumni.de (F.S. Fedorov), vsysoev@sstu.ru (V.V. Sysoev).

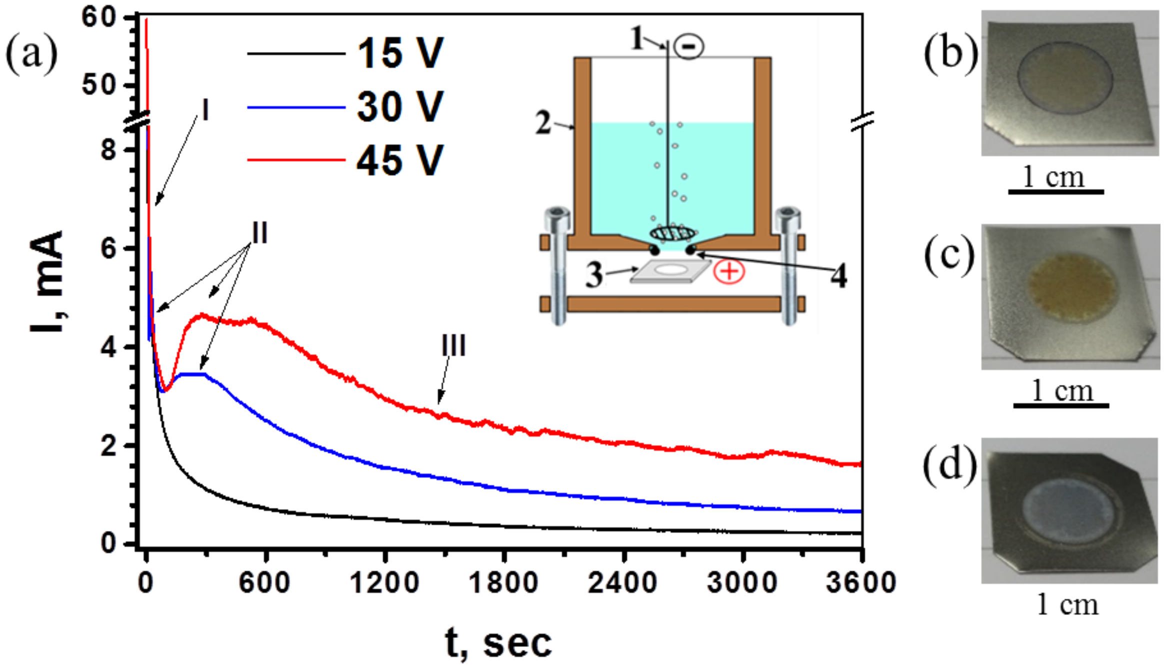

Supplementary Figure 1: The process of Ti anodization to fabricate the TiO_x NT arrays: (a) current transients at biases of 15, 30 and 45 V, insert: the electrochemical cell (scheme), 1 – cathode (Ti foil), 2 – ®Teflon cell, 3 – anode (Pt mesh), 4 – O-ring; the photos of the Ti foil following the anodization at bias equal to 15 V (b), 30 V (c) and 45 V (d).

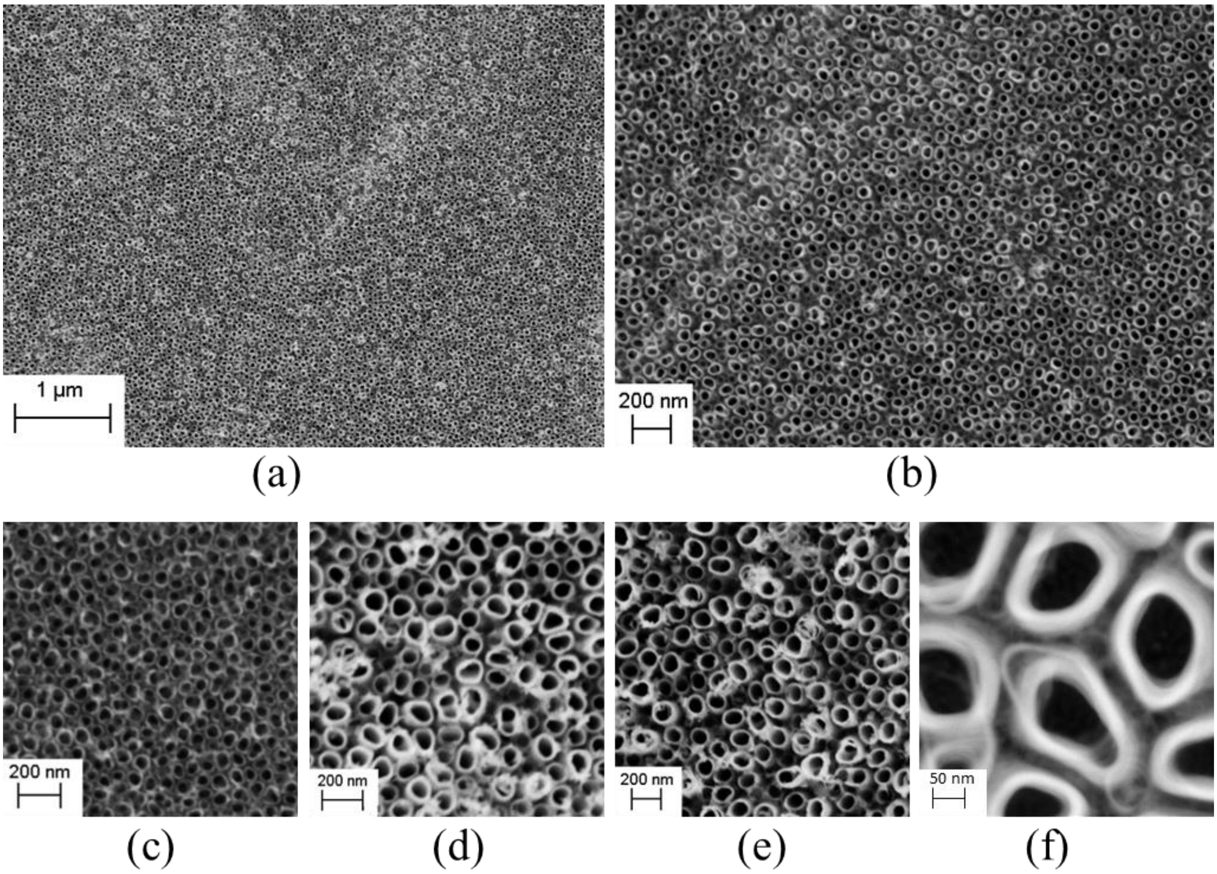

Supplementary Figure 2: Scanning electron microscopy images of TiO_x NT arrays at different magnifications. The samples have been obtained by anodization under a bias: of 15 V (a-c); of 30 V (d); of 45 V (e); HAADF-STEM image of sample obtained at 30 V (f).

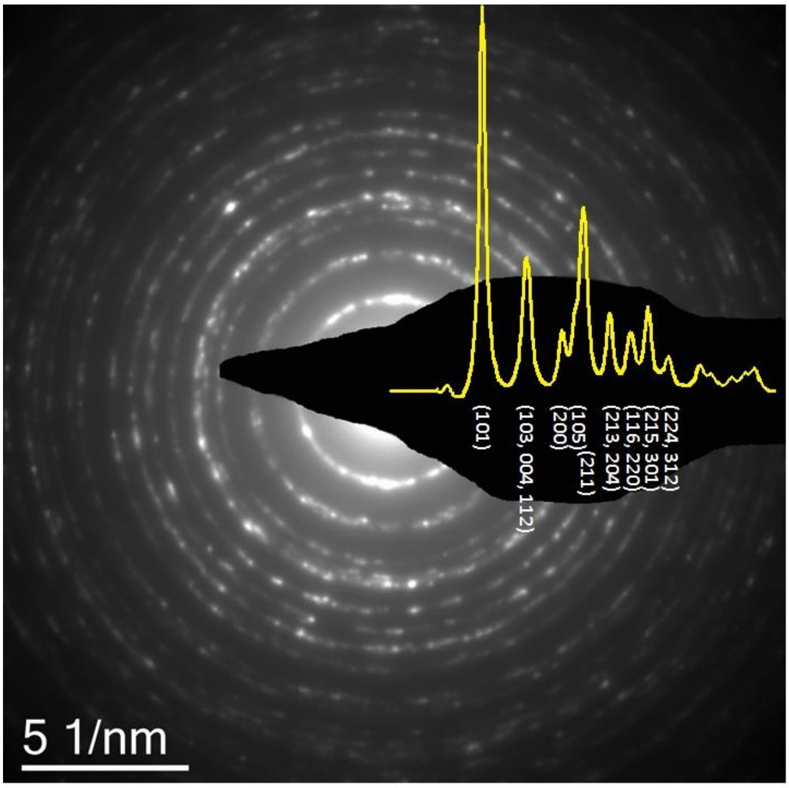

Supplementary Figure 3: SAED pattern of the annealed NT array with indexed reflections.

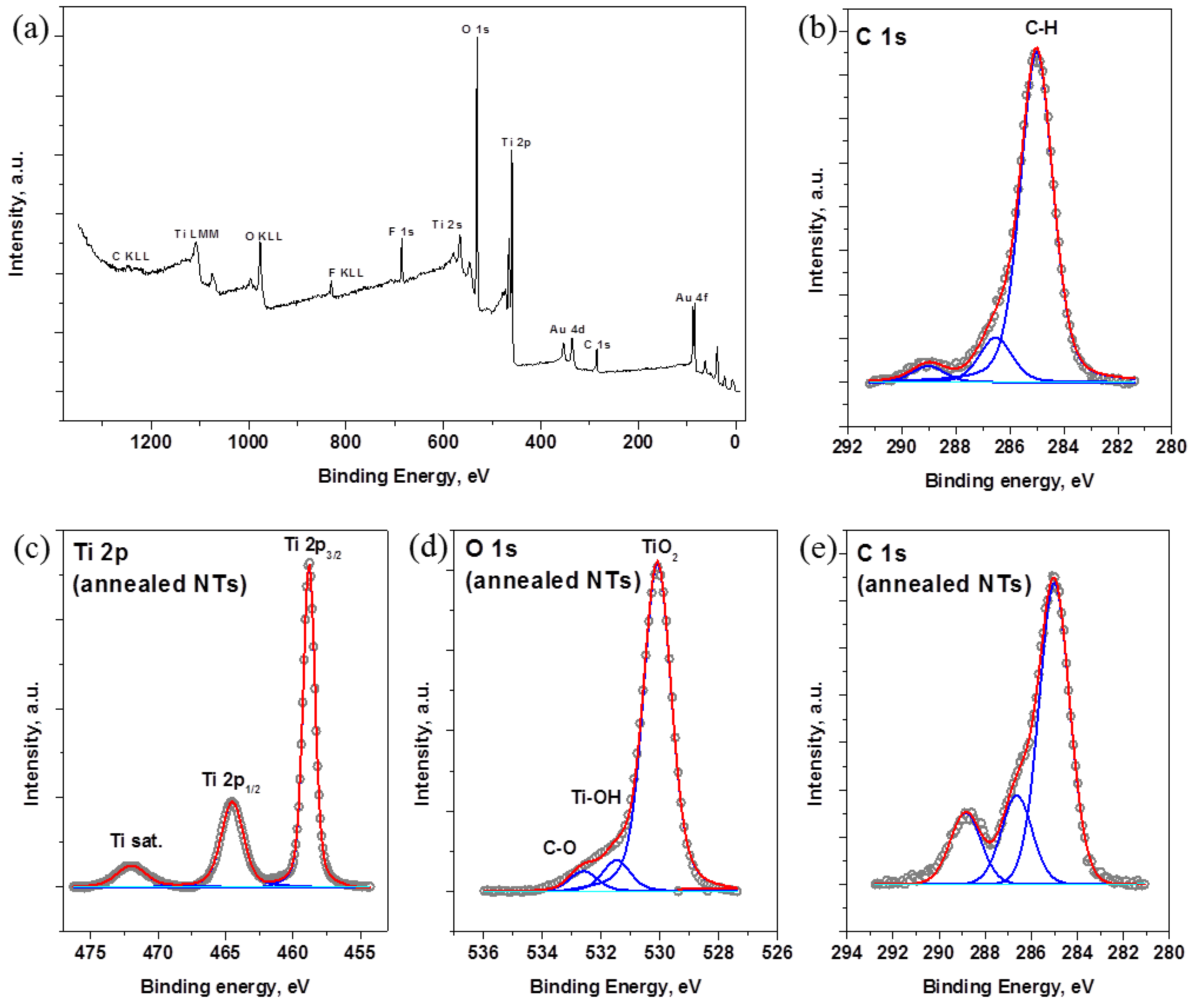

Supplementary Figure 4: XPS characterization of TiO_x NT array: (a) survey spectrum of as-prepared layer; (b) the area of XPS spectrum related to C 1s in as-prepared sample; (c-e) the areas of XPS spectrum related to Ti 2p, O 1s and C 1s of the annealed samples, correspondingly. Blue lines denote calculation results which are employed to fit the experimental curves. Red lines show the envelope curves of all fitted peaks to compare with the experimental data (open circles).

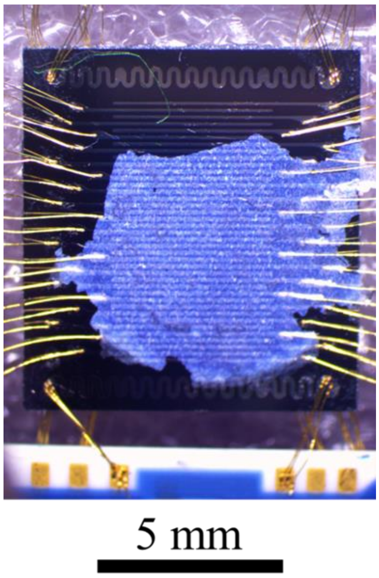

Supplementary Figure 5: The photo of multisensor chip based on TiO_x NT array which has been fabricated by anodization at bias of 30 V.

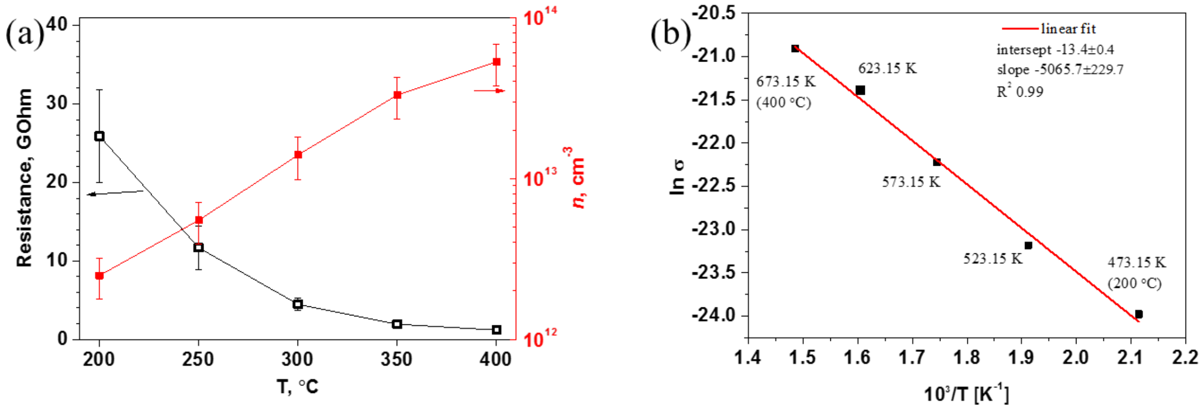

Supplementary Figure 6: The resistance of TiO_x NT array segments of the multisensor chip (black points) and free carrier concentration (red points) in dependence on operating temperature, 200-400 °C (lines are just guide for an eye), recorded in air. (a) – the original data; the error bars account for all the studied segments in the chip; (b) Arrhenius plot corresponding to the resistance *versus* temperature dependence.

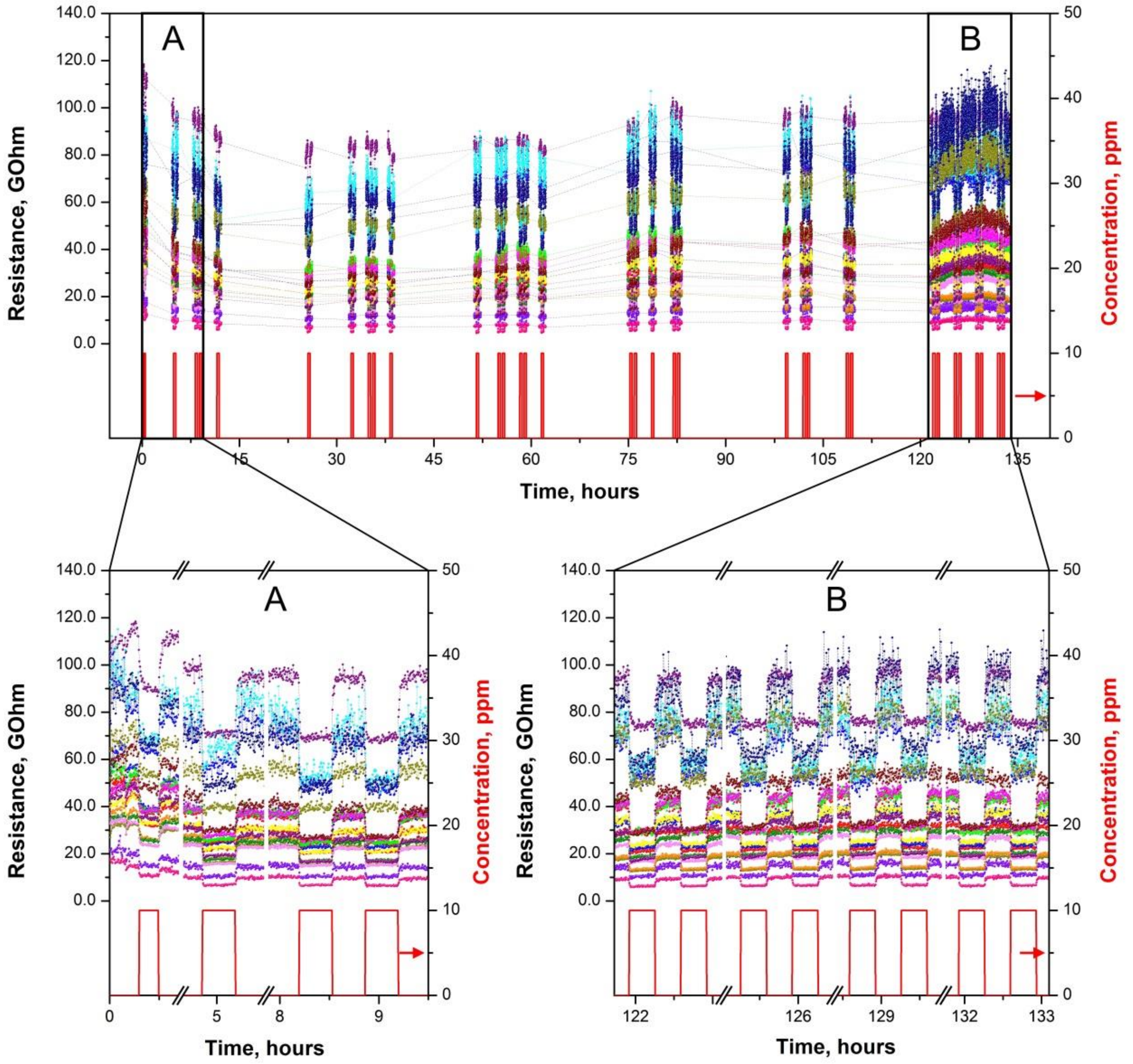

Supplementary Figure 7: The *R(t)* curves recorded with TiO_x NT –based chip under pulsed exposure to isopropanol, 10 ppm concentration, in mixture with lab air, of 30-35 rel. % humidity, during 6 days of continuous operation at 350 ^oC, starting after a fabrication and primary annealing. These data belong to another chip prepared in the same way like one described in the manuscript.

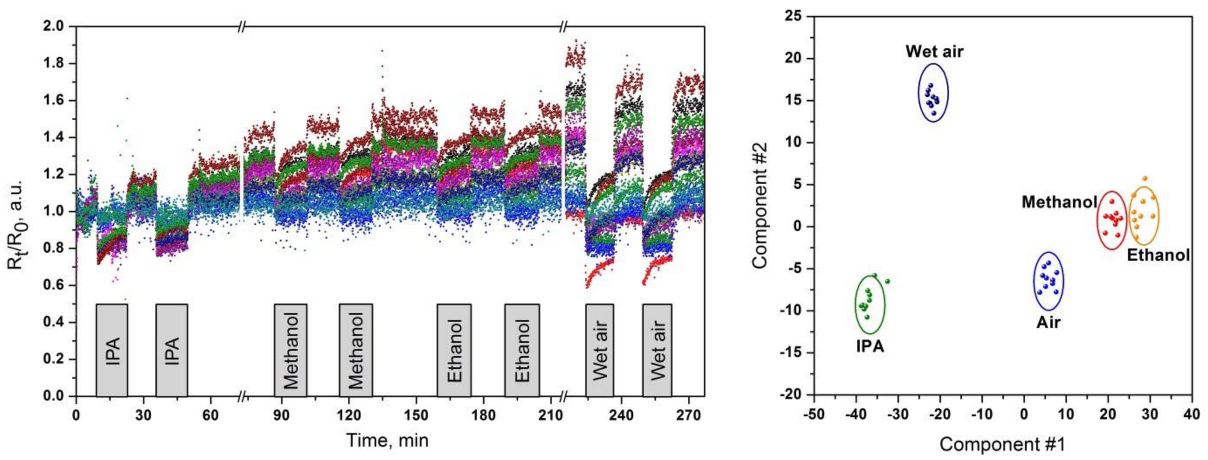

Supplementary Figure 8: Left: the normalized *R(t)* curves recorded with TiO_2 NT –based chip under pulsed exposure to isopropanol (IPA), methanol, ethanol, all at 10 ppm concentration, in mixture with lab air of 30-35 rel.% humidity, and highly humidified air, up to 100 rel. % ("wet air"). Right: the LDA processing of the response data. The data belong to another chip prepared in the same way like one described in the manuscript.

Supplementary Table 1: The d-values acquired from SAED data recorded for annealed NTs versus reference ones known for anatase TiO_2 phase

d-values$_{exp}$ [Å]	d-values$_{Anatase}$ [Å]*	crystal plane index
3.52	3.52	101
2.37	2.37	103, 004, 112
1.89	1.89	200
1.75	1.7	105
1.68	1.67	211
1.48	1.49	213, 204
1.35	1.36	116, 220
1.26	1.25	215, 301
1.17	1.16	224, 312

-

* ICSD 9852

Supplementary Note 1. Details of experimental setup and Sample characterization

A. Sample preparation

Current transients recorded during the Ti anodization under various biases, 15-45 V, are drawn in Supplementary Figure 1a. All the *I(t)* curves have a similar behavior. The current magnitude increases with the magnitude of the applied bias which is rather a common feature[1]. The *I(t)* curves consist of three clearly distinguished regions. The first (I) region related to reduction of current corresponds to a growth of oxide phase at the start of anodization process[2]. In the second (II) region the current goes up due to some dissolution of TiO_2 phase and formation of nanopores which are prerequisites for further growth of nanotubes. The next (III) stage is characterized by equilibrium between the formation and dissolution of oxide phase. The photos of samples of TiO_2 NT arrays obtained under various biases are presented in the Supplementary Figure 1 (c-d).

B. Scanning electron microscopy measurements

Scanning electron microscopy images and transmission electron microscopy images show a homogeneous layer of TiO_2 NTs with different density, diameter and wall thickness (Supplementary Figure 2). As one can see from the figure, the increase of applied bias during the Ti anodization leads to enhancing the NT diameter and reducing the density of NT array.

C. Material characterization by TEM/SAED and XPS

The SAED results for the NT array annealed at 400 °C are drawn in the Supplementary Figure 3. The obtained d-values of the NT array are listed in the Supplementary Table 1 to be compared with reference data. The obtained d-values correspond well to anatase TiO_2 phase.

An overview XPS scan drawn in the Supplementary Figure 4a indicates a presence of several elements in as-anodized sample. Besides titanium, oxygen and carbon we observe peaks attributed to fluorine. The fluorine traces appear due to its sorption on the surface of TiO_x NT wall under anodization in fluorine-

containing electrolyte[1]. Annealing of the NT array at 400 °C does not greatly affect their qualitative chemical composition. The peak O 1s due to adsorbed organic species is at 532.6 eV for the obtained anatase NTs which corresponds to 288.9 eV of the C 1s. No fluorine traces are noted in the XPS survey of the annealed sample that means its removal possibly into gaseous phase.

D. Chip overview

The Supplementary Figure 5 depicts the photo of the multisensor chip based on TiO_x NT array prepared by anodization at 30 V bias. The NT array layer has been separated from Ti and transferred into the multielectrode chip by pulling the layer out from the water surface analogously to Langmuir-Blodgett approach.

Supplementary Note 2. Sheet resistance and Activation energy

A. Sheet resistance

Sheet surface resistance per square, R_s, of the NT array is calculated as $R_s = R_{mean}\frac{l}{w}$, where R_{mean} is mean measured resistance, l the interelectrode distance, 80 µm, w the electrode length, 4 mm, which frames the measured layer under application of electric field.

B. Activation energy

The Supplementary Figure 6 shows the dependence of resistance of TiO_x NT array segments in the multisensor chip on operating temperature under air conditions. The activation energy is extracted from the following equation[3]:

$$\ln\sigma(T) = \ln\sigma(0) - E_a / kT \, ,$$

where $\sigma(T)$ is semiconductor conductivity at the given operating temperature T (K), E_a the activation energy of electrical conduction, k the Boltzmann constant.

Supplementary references:

1. Kowalski, D., Kim, D. & Schmuki, P. TiO_2 nanotubes, nanochannels and mesosponge: self-organized formation and applications. *Nano Today* **8,** 235–264 (2013).

2. Roy, P., Berger, S. & Schmuki, P. TiO_2 nanotubes: synthesis and applications. *Angew. Chemie Int. Ed.* **50,** 2904–2939 (2011).

3. Mardare, D. & Rusu, G. I. Electrical conduction mechanism in polycrystalline titanium oxide thin films. *J. Non. Cryst. Solids* **356,** 1395–1399 (2010).
